# Real-time monitoring of breath methane concentration in dairy cows using tunable diode laser spectroscopy and facemask-based sampling

**DOI:** 10.3168/jdsc.2026-1086

**Published:** 2026-09-07

**Authors:** Yueyu Lin, Joris Meurs, István Fodor, Elaine van Erp–van der Kooij, Yvette de Haas, Simona M. Cristescu

**Affiliations:** 1 Trace Detection Laboratory, Institute for Molecules and Materials, Radboud University, Nijmegen, 6525 AJ, the Netherlands; 2 Animal Breeding and Genomics, Wageningen Livestock Research, Wageningen University & Research, Wageningen, 6708 PB, the Netherlands; 3 HAS Green Academy, 's-Hertogenbosch, 5223 DE, the Netherlands

## Abstract

Accurate measurement of enteric methane (CH_4_) emissions from dairy cows remains challenging because it fundamentally relies on CH_4_ concentration measurements that are difficult to obtain accurately under farm conditions. Here, a facemask-based sampling system combined with tunable diode laser absorption spectroscopy was developed for real-time monitoring of CH_4_ concentration in exhaled breath of individual cows. The system revealed distinct dynamic CH_4_ concentration patterns characterized by baseline respiration and transient peaks associated with eructation events, with concentrations reaching up to 8,000 ppm by volume. Peak concentrations were typically 4 to 10 times higher than baseline levels, with consistent minimum/maximum ratios of 0.1 to 0.3 across animals. The proposed approach provides new insights into methane release dynamics and offers potential for improved emission quantification through integration with flow measurements.

## Graphical Abstract

**Figure:**
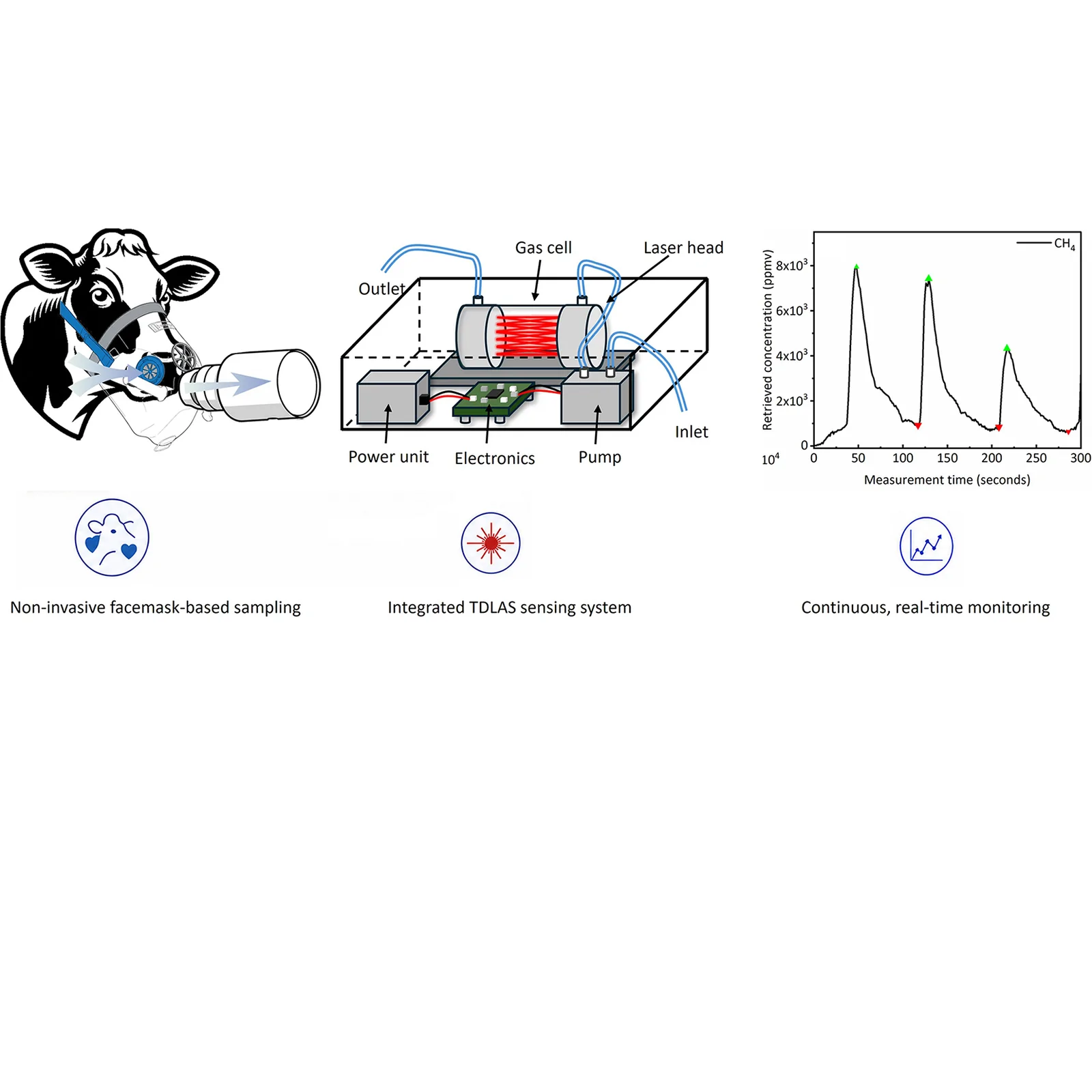

Global climate change is a major environmental concern driven largely by GHG emissions (Mikhaylov et al., 2020), with carbon dioxide (CO_2_), methane (CH_4_), and nitrous oxide (N_2_O) being the primary contributors to atmospheric radiative forcing (Shurpali et al., 2019). Agriculture accounts for approximately 9% to 14% of anthropogenic GHG emissions (Pascuzzi et al., 2024; Sokal and Kachel, 2025), with ruminant livestock, through enteric fermentation, representing a major source of CH_4_ within this sector. Although CH_4_ has an atmospheric lifetime of only ∼12 years, its warming potential is 28 times that of CO_2_ (IPCC, 2014). Therefore, accurate monitoring of CH_4_ emissions from ruminants is essential for understanding emission dynamics and developing effective mitigation strategies.

Enteric CH_4_ is produced during the anaerobic fermentation of feed in the rumen and is primarily released through eructation and exhalation via the mouth and nostrils (Dougherty et al., 1964; Dougherty, 1968; Zhao et al., 2020). A number of techniques have been developed to measure enteric CH_4_ emissions, including climate respiration chambers (**CRC**; Derno et al., 2009; Machado et al., 2016), the sulfur hexafluoride (SF_6_) tracer technique (Johnson et al., 1994; Pinares-Patiño et al., 2011; Lassey, 2013), GreenFeed (**GF**; C-Lock Inc., Rapid City, SD) systems (Hristov et al., 2015; Hammond et al., 2016; McGinn et al., 2021), the sniffer method (Madsen et al., 2010; Garnsworthy et al., 2012; Difford et al., 2018), and ventilated hood systems (Place et al., 2011; Castelán Ortega et al., 2020). Climate respiration chambers are widely regarded as the gold standard for quantifying total CH_4_ production from both rumen and hindgut fermentation. However, CRC systems are expensive and lack portability due to their size and infrastructure requirements. In addition, housing animals in enclosed chambers may induce stress and alter their natural behavior. The sulfur hexafluoride (SF_6_) tracer technique requires the placement of a permeation bolus in the rumen, which raises concerns regarding animal welfare and management complexity. Alternative approaches, including the sniffer method and ventilated hood systems, estimate CH_4_ emissions by measuring exhaled breath in barn environments. These methods are often challenged by dilution effects from ambient air and variability in the distance between the animal and the sampling inlet, which can affect measurement accuracy. GreenFeed represents a methodologically distinct approach: rather than passively sampling ambient breath, it uses a controlled airflow system to actively capture exhaled gases and directly calculate CH_4_ and CO_2_ fluxes from simultaneous concentration and airflow measurements, in short-duration visits throughout the day, conceptually similar to an intermittently operated open-circuit respiration chamber. As fluxes are calculated directly, GF is considerably less sensitive to dilution effects than concentration-based sniffer or ventilated hood systems. Facemask (**FM**) systems are an alternative approach for measuring enteric CH_4_ emissions by directly capturing exhaled breath from the animal. In this approach, a mask is fitted over the muzzle and secured to the head or neck, enabling close-range sampling of respiratory gases. A key advantage of FM techniques is the ability to measure exhaled CH_4_ from individual cows with minimal dilution from ambient air, thereby improving measurement accuracy compared with open-air methods. Previous studies have demonstrated that FM-based measurements can provide results comparable to those obtained using CRC (Silveira et al., 2019) and, moreover, have demonstrated that short-term use of a facemask does not adversely affect animal welfare, including milk production, body weight, blood parameters, and behavioral responses (Damián et al., 2022). However, most existing FM-based approaches do not provide sufficient temporal resolution (sub-second scale) to capture rapid fluctuations in exhaled CH_4_ concentrations (Oss et al., 2016; Silveira et al., 2019). Laser-based spectroscopy is an alternate technique that is widely used for gas sensing because of its high sensitivity, high selectivity, and capability for real-time, nonintrusive measurements (Fathy et al., 2022; Fu et al., 2022). Among these techniques, tunable diode laser absorption spectroscopy (**TDLAS**) has been extensively applied for trace gas detection (Sun et al., 2013) and was successfully applied for monitoring the exhalation profiles of CH_4_ and CO_2_ in human subjects (Lin et al., 2024). However, its application in ruminant breath is yet to be explored.

In this study, we present a facemask-based sampling system coupled with TDLAS for real-time monitoring of CH_4_ concentration in exhaled breath from dairy cows. A portable sensor based on a commercial original equipment manufacturer module (LGD Compact-A CH_4_, Axetris, Kaegiswil, Switzerland) was developed and tested using known CH_4_ concentrations to evaluate its response under controlled conditions. Afterward, we demonstrated the sensor performance for real-time CH_4_ detection in exhaled breath of dairy cows. We hypothesized that continuous, mask-based breath sampling would enable the detection of distinct, eructation-associated CH_4_ concentration peaks superimposed on a lower baseline respiratory signal, reflecting the intermittent nature of rumen-derived methane release, and that such dynamic patterns would not be expected to captured by conventional time-averaged or periodic sampling approaches.

The experimental procedures involving animals were approved by the Dutch Central Commission for Animal Experiments (approval number: AVD40100202317181). The experiment was conducted at a commercial dairy farm in the Netherlands between February 2024 and May 2024. The cows had a mean BCS of 3.1 ± 0.28, assessed using a DeLaval BCS camera; live body weight was not recorded. The mean parity was 3 ± 1.88, mean DIM was 171 ± 103, and the average herd milk production level was 10.382 ± 1.811 kg/d per cow. Lactating cows were housed together in a single group with cubicles and slatted floors. Animals were fed a TMR twice daily at the feeding fence, with concentrates provided individually via feeding boxes. During the summer period (April–November), cows had access to pasture adjacent to the farm during the day, from approximately 1100 h to 1500 h, following morning milking and feeding. The indoor climate was not artificially controlled (no heating or cooling systems were in place) and relied on natural ventilation, reflecting the moderate climatic conditions typical of the Netherlands, with occasional warm days in summer and mild winters. Data were obtained from 36 Holstein dairy cows, which were measured daily within the first 7 d after calving. A total of 215 measurements from 34 cows were included in the final analysis after rigorous data quality control. Some measurements were discarded due to operational issues during the field experiments and high noise levels. During the experiment, cows were fixed in headlocks at the feeding table for the duration of sampling and were fitted with a customized facemask to collect and temporarily store exhaled breath. The sensor was connected to the facemask, and an internal pump continuously directed a portion of the exhaled air to the sensor for analysis, where CH_4_ concentration was measured in real time. All measurements were performed directly after morning feeding, and each sampling session lasted ∼6 min to minimize stress to the cows. Concentration data were recorded using an analog-to-digital data acquisition card (USB-6008, National Instruments) at a sampling frequency of 1,000 Hz. The data acquisition (DAQ) card was connected to a computer via a USB interface, and data acquisition and storage were performed using a custom-written LabVIEW program. The actual data recording frequency was 2 Hz.

The schematic design of facemask-based sampling system is shown in Figure 1(a). A tightly fitting facemask (A; Air one, Hippomed/Neu-Tec GmbH, Steinhagen, Germany) was used and adapted for cattle. Two one-way valves (B) on both sides allow inspiration. In addition to these valves, a 25-mm vent (C) was controlled manually to minimize breath resistance for each dairy cow. A one-way valve (D) was installed in front of the facemask, where the exhalation was transferred to a 1-L polytetrafluoroethylene (**PTFE**) cylinder (E). This cylinder was connected after the front valve for temporary storage of breath gas sample. The bottle of the cylinder was opened to prevent the overpressure of the gas sample. The cylinder was refreshed by the exhalation during the measurement. The facemask and the PTFE cylinder were cleaned between cows to remove excess condensation. A 1/8-inch (3.2 mm) vent (F) was connected to the CH_4_ sensor. The flow rate of the pump in the sampler was 42 L/h to exchange the gas cell within a few seconds. Figure 1(b) shows the basic principle of TDLAS. During the experiment, the exhaled breath sample was directed from the 1/8-inch vent (F) to the gas inlet (I) and subsequently into the gas cell of the sensor. Inside the gas cell, a near-infrared laser beam emitted from the laser head (G) was reflected multiple times between 2 concave mirrors (H, K), thereby increasing the optical path length and enhancing detection sensitivity before reaching the detector (L). The gas sample flowed continuously through the gas cell and interacted with the laser beam. As the beam passed through the gas sample, part of the radiation was absorbed by gas molecules according to their unique absorption features. Based on the Beer–Lambert law, the measured absorbance was used to determine the gas concentration, as follows:

$$\text{\textbackslash{}[}I\left(\lambda \right)=I_{0}\left(\lambda \right)\times e^{-\alpha \left(\lambda \right)\times L},\text{\textbackslash{}]}$$

where *I*(*λ*) is the intensity of a laser beam received by the detector, *I*_0_(*λ*) is the original intensity of the laser beam out of the laser head, *λ* is the wavelength of the laser beam, *α*(*λ*) is the linear absorption coefficient, and *L* is the effective absorption path length through the sample.

**Figure 1:**
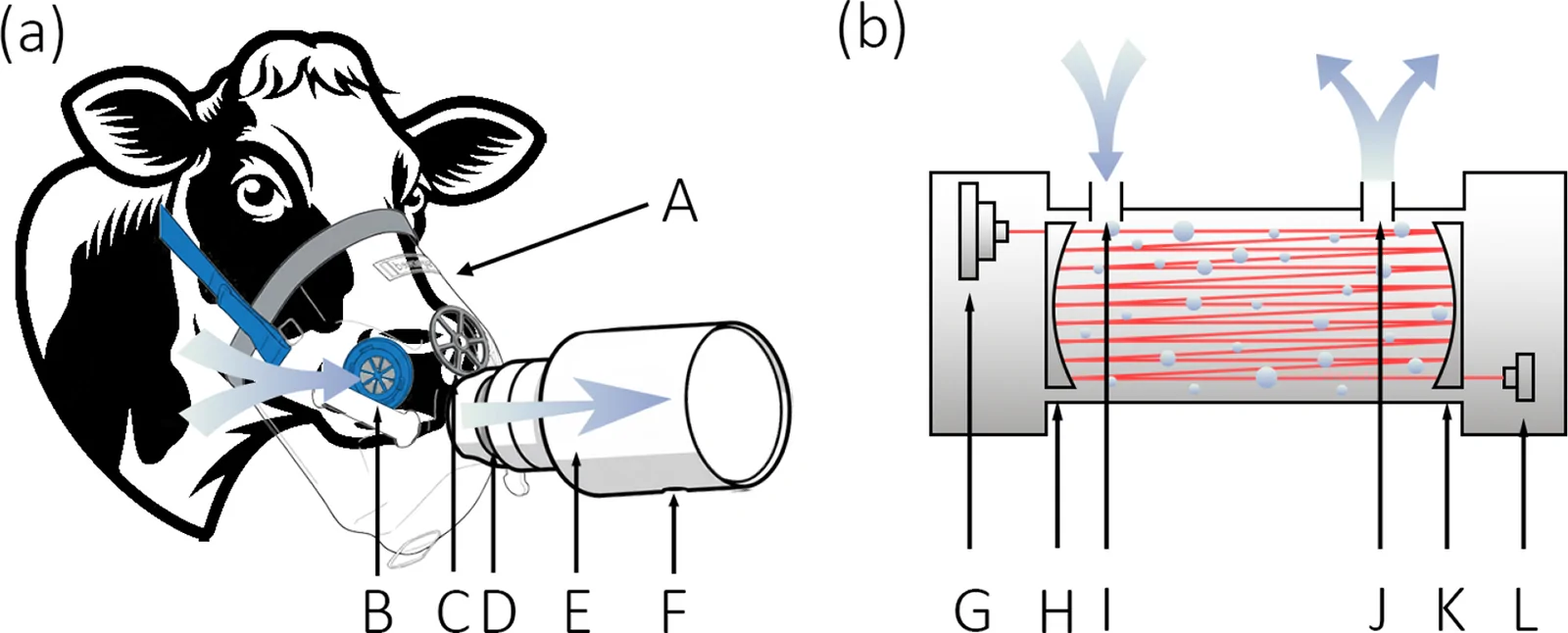
(a) The schematic design of the breath sampling system. A: Adapted facemask; B, D: one-way valves on both sides; C: 25-mm vent; E: PTFE cylinder; F: 1/8-inch (3.2 mm) vent. (b) Basic principle of tunable diode laser absorption spectroscopy. G: Laser head; H, K: optical mirrors; I: gas inlet; J: gas outlet; L: laser detector.

The sensor linearity was evaluated in the laboratory by dynamic dilutions of CH_4_ from a calibrated cylinder containing 2.17% (±2%) CH_4_ in synthetic air using 2 mass flow controllers (EL-FLOW Prestige, Bronkhorst, Ruurlo, the Netherlands). Calibration concentrations were selected to span the expected working range based on preliminary measurements (up to approximately 10,000 ppm by volume [**ppmv**]). The CH_4_ from the cylinder was diluted to 2,170, 4,340, 8,680, and 10,850 ppmv. Nitrogen was also used to tested the zero-point of the sensor. The data recorded by the custom-written LabVIEW program were replotted in Origin (2023b, OriginLab Corporation, Northampton, MA). The results are shown in Figure 2(a), where the retrieved concentrations are plotted against the applied concentrations. All individual data points (in black) are presented without averaging, and error bars represent the SD calculated from 10 consecutive measurements. A linear fit to the retrieved concentrations is plotted in red, yielding a coefficient of determination (R^2^) of 0.99 and a slope of 1.00, indicating excellent linearity and agreement between applied and measured CH_4_ concentrations. Figure 2(b) shows a real-time CH_4_ concentration profile measured from a dairy cow. The data exhibited a regular pattern characterized by local minima (marked with red triangles, 766.9 ± 146.8 ppmv) and local maxima (marked with green triangles, 6,568.8 ± 1,961.2 ppmv), occurring at a frequency of approximately one event per minute. Local minima and maxima were first identified using a simple local extrema detection algorithm in MATLAB (R2024b, The MathWorks Inc., Natick, MA). Because small fluctuations were occasionally misidentified as eructation events, the detected peaks were subsequently reviewed and manually curated to exclude false positives. Methane produced through anaerobic ruminal fermentation reaches the lungs via 2 routes: a minor fraction is absorbed through the gastrointestinal epithelium and transported in the blood, whereas the bulk is expelled during eructation, when closure of the nasopharyngeal sphincter redirects gas flow into the trachea, transiently interrupting respiratory airflow (Dougherty, 1968; Jaimes et al., 2025). This fraction of CH_4_, ∼95% (Murray et al., 1976), is subsequently released through the nostrils during subsequent exhalation. Therefore, a transient peak in CH_4_ concentration occurs after eructation, followed by a gradual decline with successive exhalations until the next eructation event. Peak CH_4_ concentrations reached up to 8,000 ppmv and are attributed to eructation events. Dynamic inter-belch CH_4_ concentration patterns have previously been described qualitatively (Koch et al., 2009) and modeled mathematically using nostril-sampled data (Jaimes et al., 2025); however, to the best of our knowledge, concentrations of this magnitude, obtained via facemask-based mouth sampling at sub-second resolution, have not been previously reported. Following each peak, the CH_4_ concentration decreased to a relatively stable baseline, primarily reflecting the background respiratory CH_4_ concentration.

**Figure 2:**
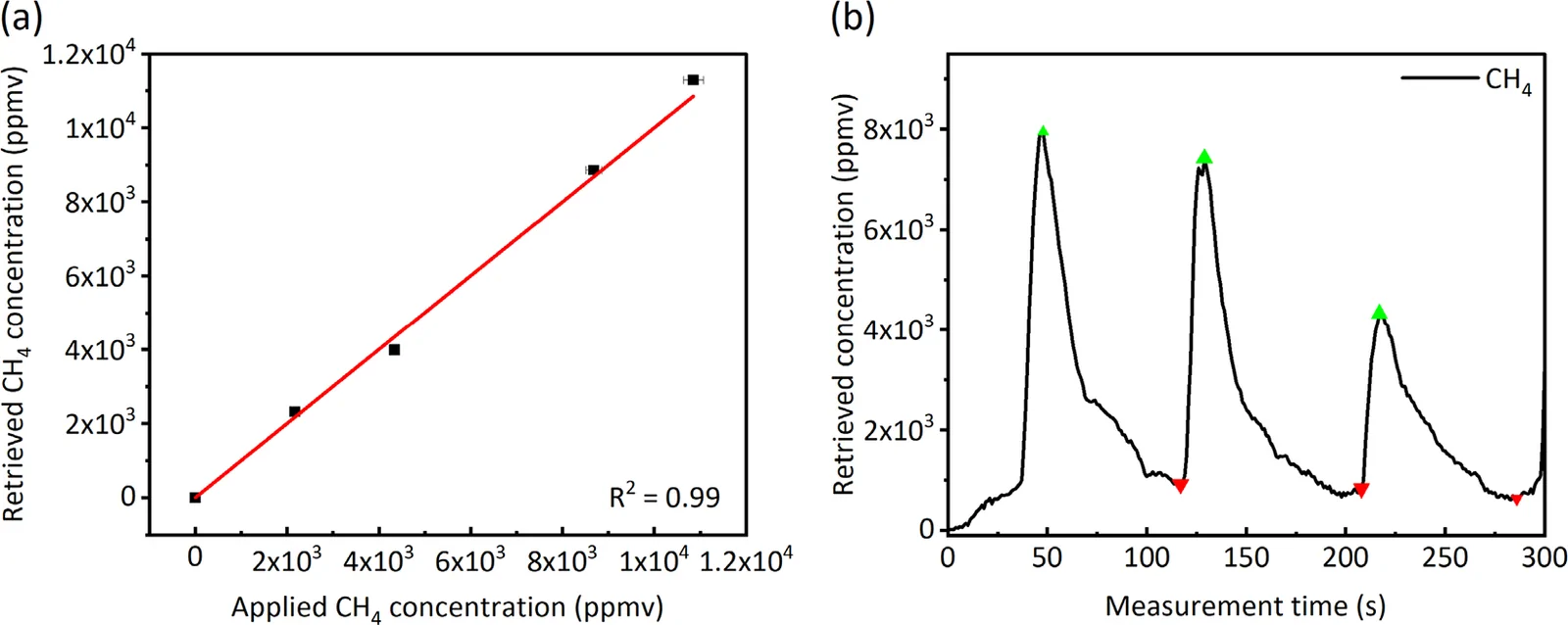
(a) Linearity of CH_4_ measurements in the laboratory (R^2^ = 0.99, slope = 1.00). (b) Real-time CH_4_ concentration profile measured on farm from dairy cows, showing peak concentrations up to 8,000 ppmv associated with eructation events. Red triangles indicate local minima and green triangles indicate local maxima.

As shown in Figure 2(b), a regular oscillatory pattern was observed during the measurements, characterized by local minima representing baseline respiration and local maxima associated with eructation events. The data presented in Figure 2(a) and 2(b) are the local minima and local maxima values of all measurements. For each cow on each day, the mean local minimum and maximum values were calculated, and normality testing was performed separately for each dataset. Figure 3(a) shows the distribution of local minimum values. The data were approximately normally distributed, with a mean of 1,012 ppmv and an SD of 303.2 ppmv. Normality testing indicated no significant deviation from normality (*P* = 0.5), suggesting a relatively stable baseline CH_4_ concentration across measurements. Figure 3(b) shows the distribution of local maximum values, with a mean of 5,366 ppmv and an SD of 1,523 ppmv. Normality testing indicated a significant deviation from a normal distribution (*P* = 0.04), likely reflecting the episodic and highly variable nature of eructation events. Local maximum values (corresponding to methane peaks) were skewed to the right. Here, h indicates the test decision (h = 0: normality not rejected; h = 1: normality rejected), and *P* represents the corresponding *P*-value. Furthermore, the ratio between corresponding local minimum and maximum values was calculated for each cow on each day. Figure 3(c) shows the mean ratio with SD for each cow across 7 consecutive days. Most ratios, including their SD, ranged between approximately 0.1 and 0.3. Two cows (numbers 26 and 32, marked in red) exhibited higher ratios exceeding 0.4, likely due to relatively lower peak CH_4_ concentrations during eructation events. Overall, these results indicate that CH_4_ concentrations during baseline respiration are typically 70% to 90% lower than those observed during eructation events.

**Figure 3:**
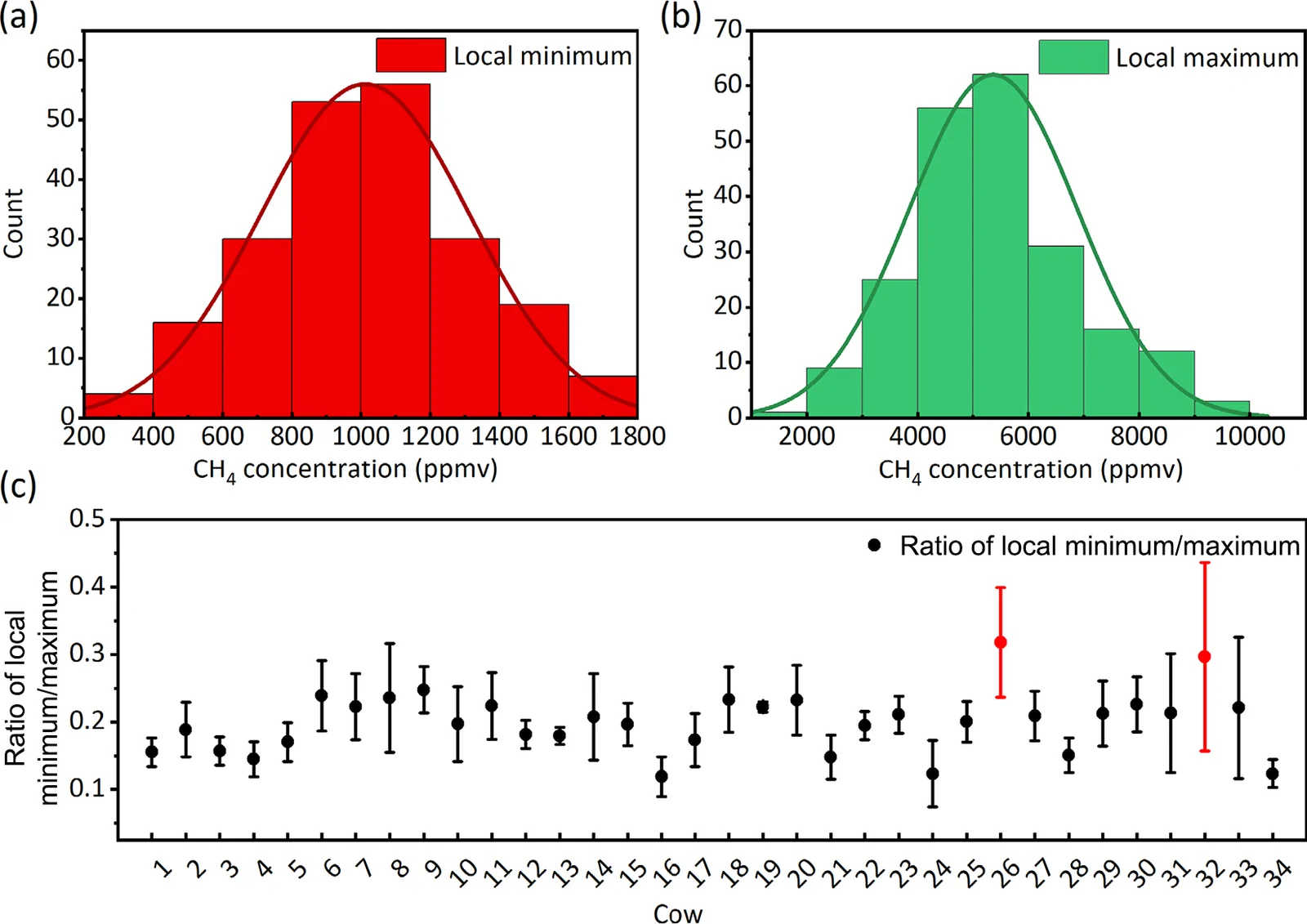
(a) Local minimum values were normally distributed (normality test: *P* = 0.5). (b) Local maximum values deviated from normal distribution (normality test: *P* = 0.04). (c) The mean ratio with SD for each cow across 7 consecutive days.

We have illustrated that real-time monitoring of breath CH_4_ using a facemask-based TDLAS system can provide detailed insight into the temporal structure of CH_4_ release in dairy cows, consistent with our hypothesis that continuous, mask-based sampling would reveal distinct eructation-associated CH_4_ peaks superimposed on a lower baseline signal. Given the benefit of high temporal resolution (sub-second level), we revealed a clear dynamic pattern in CH_4_ concentration, characterized by baseline respiration and intermittent peaks associated with eructation events. The observed separation between local minima and maxima, together with the consistent minimum/maximum ratio (typically 0.1 to 0.3), indicates that CH_4_ release is dominated by short-duration, high-intensity events. The result is further supported by the distinct statistical behavior of the 2 components, where baseline concentrations showed a relatively normal distribution, whereas peak values exhibited greater variability and deviation from normality, reflecting the episodic nature of eructation. The facemask incorporates several design features that support its use under farm conditions. One-way valves allow the animal to breathe freely with minimal resistance, and the mask is designed to minimize dilution of exhaled breath with ambient air by sampling directly at the animal's nose/mouth, thereby improving the accuracy of CH_4_ concentration measurements. The system is also portable (approximately shoebox-sized) and relatively low cost (in the order of thousands of euros), facilitating deployment in on-farm or research settings. Compared with GF, which relies on the animal voluntarily visiting a feeding station and therefore provides only intermittent, visitation-dependent measurements, the facemask enables continuous, uninterrupted sampling of exhaled breath regardless of the animal's location or behavior, allowing detection of dynamic CH_4_ patterns, such as individual eructation events. However, a key limitation of the facemask-based system should be considered for its use at the farm or research level. The current implementation requires an operator to fit the mask on each animal and does not allow for the unattended, high-throughput monitoring achieved by systems such as GF, respiration chambers, or sniffer-based sensors.

Some conventional methods that rely on time-averaged measurement (e.g., CRC) cannot resolve such dynamic features. In contrast, the real-time CH_4_ measurements presented here can serve as a trigger signal to identify transient emission events. This enables differentiation between baseline respiratory signals and rumen-derived gas released during eructation, although complete separation of these components is not feasible under practical conditions (Barrientos-Blanco et al., 2025). However, it should be acknowledged that the present system requires substantially greater human labor input than established large-scale approaches such as GF, respiration chambers, and sniffer-based sensors, which already enable unattended, high-throughput monitoring across large herds without requiring an operator to be present, which is a considerable practical advantage for emission monitoring at scale. The comparative strength of the present system therefore lies primarily in continuous, individual-level health surveillance rather than in large-scale emission quantification. Combining continuous CH_4_ concentration monitoring with other physiological parameters may, in principle, offer a noninvasive complement to existing rumen-monitoring approaches in future work, although this direction is supported only by preliminary evidence in the literature (Antanaitis et al., 2022) and requires dedicated validation in this system. However, further research is also required to validate our sensor, including larger-scale studies, longer-term monitoring, and integration of flow measurement. In addition, the portability (approximately shoebox-sized) and relatively low cost (in the order of thousands of euros) of the proposed system may offer advantages for CH_4_ monitoring in specific research or individual-animal contexts, such as validating or complementing existing large-scale approaches. The facemask-based sampling design minimizes dilution from ambient air, thereby improving the accuracy of breath CH_4_ measurements. By integrating a flow sensor into the sampling system (e.g., within the PTFE cylinder), the volumetric flow rate of exhaled breath can be measured, enabling conversion of CH_4_ concentration (ppmv) into an estimated emission rate (e.g., g/d). However, accurately measuring airflow under the dynamic conditions of facemask application, including rapid airflow fluctuations, mask leakage, and transient breathing patterns, remains an unvalidated technical challenge requiring future work. Inspired by the manual baseline-peak distinction approach used by Caetano et al. (Caetano et al., 2018) to separate rumen- and lung-derived CO_2_ in GF data, applying a similar framework to continuous breath CH_4_ data for quantitative partitioning of baseline respiratory signal and eructation-derived CH_4_. However, obtaining representative daily CH_4_ production requires repeated measurements to account for diurnal variation associated with feeding behavior, as methane emissions typically increase after feeding and decrease until the next feeding event; at least 7 to 8 measurements across a 24-h period are recommended to adequately capture this temporal variability. (Pressman et al., 2025). This repeated-measurement requirement represents a substantial labor demand and is a key factor currently limiting the practicality of the system for large-scale, on-farm emission monitoring, in contrast to established unattended approaches such as GF and respiration chambers. Future development of automated mask-fitting and data acquisition procedures may help reduce this labor burden, although such approaches remain to be developed and validated. In this study, a facemask-based sampling system combined with TDLAS was developed for real-time monitoring of CH_4_ in exhaled breath from dairy cows. The system enables high-temporal-resolution measurements under farm conditions while minimizing dilution from ambient air. The results revealed a clear oscillatory pattern in CH_4_ concentration, characterized by baseline respiration and transient peaks associated with eructation events. Quantitative analysis showed that peak concentrations were typically 4 to 10 times higher than baseline levels, with consistent minimum/maximum ratios across animals, highlighting the event-driven nature of methane release. Furthermore, the proposed system shows possibility for emission quantification by integrating concentration and flow measurements, although flow measurement remains an unvalidated technical challenge requiring future development. Combining continuous CH_4_ concentration monitoring with other physiological parameters may, in principle, offer a noninvasive complement to existing rumen-monitoring approaches in future work, although this direction is supported only by preliminary evidence in the literature (Antanaitis et al., 2022) and requires dedicated validation in this system. Overall, this work provides a practical and informative approach for advancing methane measurement in dairy production systems.
